# Treatment of unstable distal radius fractures with the volar locking plate

**DOI:** 10.3109/03009734.2011.594183

**Published:** 2011-10-29

**Authors:** Hanae Minegishi, Osamu Dohi, Soukan An, Hidetsugu Sato

**Affiliations:** ^1^Department of Orthopedic Surgery, Tohoku Kosai Hospital, Sendai, Japan; ^2^Ayashi Orthopedic Clinic, Sendai, Japan; ^3^Sato Hidetsugu Orthopedic Clinic, Sendai, Japan

**Keywords:** Complications of locking plate, distal radius fracture, internal fixation, volar locking plate

## Abstract

**Background:**

Open reduction and internal fixation using an interlocking plate system has gained popularity for the treatment of dorsally displaced distal radius fractures.

**Purpose:**

To evaluate the functional and radiological results of treating unstable distal radius fractures with the volar locking plate.

**Patients and methods:**

A retrospective review was conducted of patients from one institution using the volar locking plate to treat intra-articular and extra-articular distal radius fractures. Unstable distal radius fractures in 15 patients, comprising 3 men and 12 women with a mean age of 64.4 years (34–76 years), were treated with a volar locking compression plate (Acu-Loc distal radius plate system; Acumed, Oregon, USA) and followed up for a minimum of 1 year. Fractures were classified using the AO classification. Radiographic parameters of preoperative, postoperative, and final follow-up radiographs were compared. The time to initiation of active range of motion was determined. Final follow-up range of motion and complications were reported.

**Results:**

At final functional assessment, the scores of 5 patients were excellent, 7 patients good, and 3 patients fair according to Cooney's Clinical Scoring Chart. No non-union or infection occurred. Rupture of the flexor pollicis longus tendon occurred in 1 patient.

**Conclusion:**

Treatment of unstable distal radius fractures with a volar locking plate leads to satisfactory results, provided the operative technique is carefully performed to prevent complications.

## Introduction

Open reduction and internal fixation using an interlocking plate system is a valid treatment of displaced extra-articular and intra-articular distal radius fractures in adults (1–4). The goals of treatment are to achieve anatomic fracture union, facilitate early range of motion, and avoid complications. When a dorsal approach is used, extensor tendon rupture and irritation caused by implants or surgical intervention are serious complications (5,6,18). Alternative treatment options include bridging external fixation and non-bridging external fixation. A volar approach has been developed to fix a dorsally angulated fracture of the distal radius. It has several advantages, including the broader volar aspect of the distal radius, the avoidance of both dorsal dissection and attendant complications of the extensor tendons, and the possible deprivation of blood supply to the dorsal metaphyseal fragments (7–9). The locking plate and its screws act as a single unit to hold the bone fragments, compared to a traditional plate which needs compression between the plate and the fragment to gain friction. This is particularly useful in the prevention of secondary displacement of the unstable fracture, especially in elderly patients with osteoporotic bones (7,8,10). The purpose of this report is to evaluate the functional and radiological results of treating intra-articular and extra-articular distal fractures with a volar locking plate.

## Patients and methods

**Figure 1.**
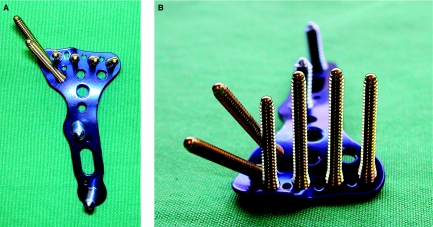
A: The distal fixed-angle screws are angled 6 degrees distally; precontoured plates are used for the volar distal radius. B: This plate has a unique screw hole for insertion at the radial styloid, and the thickness of the plate is 2.5 mm.

Fifteen patients with distal radius fractures were treated with the Acu-Loc distal radius plate (Acumed) in the Department of Orthopedic Surgery, Tohoku Kosai Hospital, Japan, from April 2007 to March 2009. The distal fixed-angle screws in the plate are angled 6 degrees distally, precontoured plates are used for the volar distal radius, and the thickness of the plate is 2.5 mm. This plate has a unique screw hole for insertion at the radial styloid (Figure 1). Exclusion criteria included any fracture treated with another volar plate or distal radius fractures extending to the shaft of the radius, and concomitant fractures of the same limb.

This study included 3 men and 12 women with an average age of 64.4 years; 5 patients had fractured their dominant wrist, and 10 patients fractured their non-dominant wrist. Altogether 11 fractures were caused by simple falls, 1 occurred following a fall from a height, 1 while skiing, 1 from a motorcycle accident, and 1 while dancing socially.

Fractures were classified using the AO classification system (11): 1 fracture was extra-articular (AO-type A) and 14 fractures were complete intra-articular (AO-type C). There were no partial intra-articular fractures (AO-type B) (Table I).

**Table I.** Classification of distal radius fractures according to the AO classification system.

Extra-articular, *n* = 1	Intra-articular, *n* = 14			
A3	B	C1	C2	C3
1	0	3	5	6

Preoperative, immediate postoperative, and final follow-up volar tilt, radial inclination, and ulnar variance were measured radiographically. Subjective and objective functional results were graded using modification of the demerit point system of Saito and Cooney's scoring system (12). Subjective evaluation consisted of pain, disability, lassitude of wrist, restricted activity, and limitation of motion. Objective evaluation consisted of range of motion of the wrist and forearm, grip power, and complications such as nerve injury, finger contracture, tendon irritation, and development of osteoarthritis.

All procedures were performed under general anesthesia with upper arm tourniquet. The distal radius was exposed by a palmar approach along the flexor carpi radialis tendon. After release of the pronator quadratus muscle from its radial insertion, the fracture site and palmar surface of the distal radius were exposed. Fracture reduction was verified with the image intensifier. Provisional Kirschner wires were used occasionally.

We usually used a condylar stabilizing technique (13). In the condylar stabilizing technique, the plate and drill guide/K-wire or locking pin construct is called the ‘reduction maneuver’. The reduction maneuver is pushed up and rotated, so that each articular fragment is reduced, and the clamp is used to maintain reduction. The reduction maneuver is lined up with the radial shaft, and then the fracture is reduced in anatomical position (epiphyseal fixation first, reduction second). Next, the length of the radius is adjusted for proper ulnar variance by pushing the plate distally to restore the distal radio-ulnar congruity and to apply adequate tension on the triangular fibrocartilage complex (TFCC). Two important objectives in this technique are to insert correctly the locking pins just beneath the mechanically reliable subchondral bone and to reduce the fracture after fixation of the epiphyseal fragments.

The pronator quadratus was repaired with 3-0 absorbable sutures. The wrist was protected with a light below-elbow splint for 2 weeks, and physiotherapy with active and passive wrist and digital mobilization out of the splint was started immediately.

## Results

The average follow-up period was 15.5 months (range 12–24 months). Artificial bone graft (tricalcium phosphate chips) was performed for 6 patients. Overall, three patients exhibited complications: two patients had slight finger contraction, and one patient suffered an attrition rupture of the flexor pollicis longus tendon, which was treated with tendon suture. There was no non-union or wound infection in this study. No breakage of plates was observed. Average preoperative, postoperative, and final follow-up radiographic parameters are listed in Table II. Preoperative volar tilt was –9.3 ± 18.8 degrees (range –40 to 28 degrees), postoperative volar tilt was a reconstructed 10.1 ± 5.8 degrees (range 0 to 25 degrees), and final follow-up volar tilt remained at 9.7 ± 5.0 degrees (range 3 to 23 degrees). Preoperative radial inclination was 14.1 ± 4.9 degrees (range 7 to 25 degrees), postoperative radial inclination was a reconstructed 19.3 ± 4.2 degrees (range 12 to 28 degrees), and final follow-up radial inclination remained at 20.0 ± 4.2 degrees (range 12 to 28 degrees). Preoperative ulnar variance was 4.3 ± 2.3 mm (range 1 to 8.7 mm), postoperative ulnar variance was a reconstructed –0.5 ± 1.4 mm (range –2 to 2.9 mm), and final follow-up ulnar variance remained at 0.2 ± 0.9 mm (range –1.7 to 2.4 mm).

**Table II.** Average (± SD) preoperative, postoperative, and final follow-up radiograph parameters.

	Preoperative	Postoperative	Final follow-up
Volar tilt, degrees	–9.3 ± 18.8	10.1 ± 5.8	9.7 ± 5.0
Radial inclination, degrees	14.1 ± 4.9	19.3 ± 4.2	20.0 ± 4.2
Ulnar variance, mm	4.3 ± 2.3	–0.5 ± 1.4	0.2 ± 0.9

Average final wrist range of motion was 55.5 ± 10.3 degrees extension (range 45 to 80 degrees) and 59.3 ± 17.5 degrees flexion (range 50 to 90 degrees). Average final forearm range of motion was 86.3 ± 17.2 degrees pronation (range 30 to 100) and 90.4 ± 5.9 degrees supination (range 80 to 100 degrees) (Table III). At final functional assessment, the scores of 5 patients were excellent, 7 patients good, and 3 patients fair according to Cooney's Clinical Scoring Chart (14). According to modification using Saito's valuation (15),14 patients were excellent and 1 was good. No patient experienced a change in daily activity or occupation.

**Table III.** Average (± SD) final wrist and forearm range of motion.

Wrist extension (degrees)	Wrist flexion (degrees)	Forearm pronation (degrees)	Forearm supination (degrees)
55.5 ± 10.3	59.3 ± 17.5	86.3 ± 17.2	90.4 ± 5.9

## Discussion

The primary goal in treatment of unstable fractures of the distal radius is to achieve proper reconstruction of the disrupted anatomy and allow the quick return of hand function without complications. Dorsal plate fixation is biomechanically effective in buttressing a dorsally displaced fracture of the distal radius. Osada et al. compared the biomechanical properties of dorsal and volar fracture fixation plate designs in a cadaver model (16). They reported that if the volarly placed titanium symmetry plate was used to fix a Colles-type fracture, the distal fragment of the radius would develop a dorsal angulation of about 9 degrees if early active mobilization of the fingers was initiated during the postoperative period. On the other hand, Leung et al. (8) demonstrated no statistical difference between axial loading transmission through the intact radius and a distal radius fracture fixed with a volar locking plate. In fact, the volar locking plate showed advantages over dorsal plating in the fixation of a dorsally unstable distal radius fracture.

In addition, volar plate fixation is a valuable method because of the decreased risk of inducing dorsal soft-tissue complications. The dorsal approach often needs dissection of the extensor retinaculum and sometimes resection of the Lister's tubercle. Therefore, the extensor tendons are generally exposed to mechanical attrition by the plate and screws. In the volar approach, the volar anatomy of the wrist presents an advantage over the dorsal aspect because there is more space between the volar cortex and the flexor tendons. The pronator quadratus can also sometimes act as a hedge to prevent soft tissue complications. The palmar cortex is relatively flat, and the plate is better contoured for application from this aspect rather than on the dorsal cortex of the distal radius (15).

The volar cortex of the distal radius was very often not as severely comminuted when compared with the dorsal cortex. Anatomical reduction of the palmar cortex may avoid the shortening of the radius, which is important for its restoration. In our series, final ulnar variance was reconstructed to excellent level, and resulted in a wide range of motion. The volar plate system used in our study was a locking plate system, and this must be one of the reasons for retaining good anatomical reduction.

Volar plate fixation of unstable distal fractures has been described recently (1–3,5,15,17). Our results are comparable to the final follow-up range of motion, radiological evaluation, and functional assessments presented in these recent reports. In regard to complications, Orbay and Fernandez (2) reported 1 case among 31 of dorsal tendon irritation from an excessively long peg, which was treated with hardware removal. Of nine patients with preoperative median nerve symptoms who had carpal tunnel release, the final neurologic examination showed complete resolution at the time of late follow-up. Rohit et al. (3) reported an overall 31 patients (27%) with complications. They considered tenosynovitis as a risk factor for progressive damage to the tendons and therefore included tenosynovitis as a complication. There were 17 tendon complications (57% of the total number of complications). Early hardware removal was performed in all patients who developed tenosynovitis. Among other complications, three patients suffered carpal tunnel syndrome (3%), screw loosening occurred in two patients (2%), and intra-articular screw displacement occurred in one patient (1%). Kamano et al. (1) used a volar plate in 40 dorsally displaced distal radius fractures. With regard to complications, the authors reported that one patient developed a complex regional pain syndrome, and another had a mild skin infection. There was no need for plate removal and no implant failure. In our study, rupture of the flexor pollicis longus tendon was observed in one patient. We could salvage this patient by end-to-end suture, but this rupture often requires tendon transfer (15,17). Early plate removal should be performed to avoid this complication. Flexor and extensor tendon irritation has also been reported in other studies as the most frequent problems (3,17).

The optimal placement of the distal screws is important: they must be inserted at the radial styloid, beneath the lunate facet, and near the sigmoid notch (18). Therefore, the plate is positioned near the volar radius margin. But fixation implants placed over or distally to the watershed line can exert pressure on the flexor tendons and cause injury (3,19). The watershed line is defined as a transverse ridge that closures the concave surface of the volar radius distally. Distal to this line, the radius slopes in a dorso-distal direction and becomes prominent palmarly (3). The course of the flexor pollicis longus tendon is close to the palmar rim of the distal radius. The plate placed very close to the wrist joint can support the palmar aspect of the articular surface. However, it sometimes causes flexor tendon impairment. In the very distal area, it is not possible for the reattached pronator quadratus muscle to protect the flexor tendons. As a result, the tendons can abrade against the plate and sharp edges of the screw heads. It also must be emphasized that protruding screw heads can cause tendon irritation (3). To avoid rupture of the flexor pollicis longus tendons, care has to be taken especially in very distal fractures, type C3 fractures, and osteoporotic bone. Adequate image intensifier control to verify the extra-articular and subchondral position of screws and plate is also quite important. In our study, a patient with rupture of the flexor pollicis longus tendon possessed an implant that was placed distally to the watershed line, and secondary displacement with loosening of the distal screws was observed. If fracture instability demands distal placement of hardware, close follow-up investigations and hardware removal should be considered at the first sign of flexor tendon irritation as reported by Drobetz and Kutscha-Lissberg (17). This was also an important point for our study.

The Acu-Loc distal radius plate is useful for achieving good anatomical reduction, but care must be taken to avoid the complication of tendon rupture. Placing the plate proximally to the watershed line and removing the plate as soon as the fracture united were necessary to avoid the complication of tendon rupture. By these considerations, volar distal radius plates will provide excellent results in treating distal radius fractures.
